# Uncertainty-Aware Training for Ophthalmic Segmentation Using MedSAM

**DOI:** 10.1167/tvst.15.2.19

**Published:** 2026-02-17

**Authors:** Christopher William Clark, Scott Kinder, Giacomo Nebbia, Yoga Advaith Veturi, Steven McNamara, Niranjan Manoharan, Talisa Forest, Malik Kahook, Naresh Mandava, Praveer Singh, Jayashree Kalpathy-Cramer

**Affiliations:** 1 University of Colorado School of Medicine, Aurora CO, USA

**Keywords:** computer vision, uncertainty quantification (UQ), glaucoma, geographic atrophy (GA), age-related macular degeneration

## Abstract

**Purpose:**

Uncertainty quantification (UQ) has been applied to deep learning (DL) models to enhance not only their interpretability, but to guide model learning. This work presents Uncertainty-Aware Training (UAT), which generates uncertainty maps at train time and augments the loss function with them to allow the models to concentrate on areas of high uncertainty during learning. By highlighting uncertain pixels as requiring more scrutiny, model performance is enhanced on three ophthalmic segmentation tasks.

**Methods:**

We applied UAT to three ophthalmic segmentation, or pixel-level classification, tasks: geographic atrophy (GA), optic cup (OC), and foveal avascular zone (FAZ). UAT weighs the loss function binary cross-entropy according to uncertainty, forcing the model to focus on ambiguous areas. We experimented with output-based UQ methods, such as entropy, and post -hoc techniques like conformal prediction. UAT was evaluated on a state-of-the-art foundational model fine-tuned for these specific segmentation tasks.

**Results:**

The addition of an entropy-weighing map at loss calculation time consistently improved model performance across all three datasets, with conformal prediction using the Least Ambiguous Set-Valued Classifier version also achieving higher overall performance for GA and OC segmentation. The easy-to-integrate implementation of UAT proved feasible without significant training structure modifications.

**Conclusions:**

UAT enhances DL model performance and interpretability by focusing on uncertainty in addition to error, improving segmentation accuracy.

**Translational Relevance:**

UAT uses interpretable uncertainty maps to enhance DL segmentation performance. Its lightweight integration makes it practical for adoption and leads to improved model performance on ophthalmic segmentation tasks.

## Introduction

One of the more difficult aspects of using a deep learning (DL) model is their general “black-box" nature, which makes it difficult to understand why the models make their decisions. To aid in model understanding, we can apply uncertainty quantification (UQ) methods. These methods can inform us of model certainty, aiding in understanding the model itself, or providing more data for a clinical decision. In this work, however, we apply these concepts not at the end or at decision time, but during training. In addition to standard supervised learning, wherein the model outputs are compared to the ground truth and the loss results in updated weights, we can provide uncertainty metrics in real time during training and force the model to consider uncertain data with more scrutiny by modifying this loss function.

We apply these methods to three ophthalmic segmentation tasks. In a segmentation task, we are interested in not just classifying an image, but highlighting parts of the image as belonging to the structure of choice. In this case, we have a geographic atrophy (GA), optic cup (OC), and foveal avascular zone (FAZ) segmentation task, and so the models will output segmentation maps indicating which pixels belong to these anatomic structures. By applying UQ during training, we produce uncertainty maps and use these to weight the loss, which we refer to as uncertainty-aware training (UAT). A high-uncertainty pixel will count more toward the loss than a certain one regardless of accuracy, and this forces the model to concentrate on highly uncertain areas in addition to incorrect ones. The objective of this work is to explore light-weight UQ pipelines integrated directly into training to improve segmentation accuracy.

We experiment with a probability-level UQ method, entropy, a Monte Carlo-like UQ method based on the variance of model predictions, and a more recent post hoc method conformal prediction.^1^^,^^2^ We test these methods on a state-of-the-art foundational model fine-tuned for these three ophthalmic segmentation tasks.

We find that, in all datasets, entropy-based UAT improves overall model performance, with the conformal prediction algorithm Least Ambiguous Set-Valued Classifier also outperforming baseline models for GA and OC segmentation. Further, although it does not reach statistical significance, the addition of entropy-based loss weighing reduces variance in model performance (i.e. increases training stability). In addition, the saved uncertainty maps allow us to understand model behavior as training progresses. Practically, each of these pipelines are light-weight and easy to implement, so adding these during experimentation should not be too difficult and, based on this work, will result in improved performance.

## Related Works and Novelty

Uncertainty in machine learning can be divided into epistemic and aleatoric, and attempts to quantify each of these uncertainty types results in unique UQ methods not found in standard statistical approaches.^3^ Although these methods can be used for any machine-learning task, they are particularly useful in medical settings where the consequences of an incorrect action can be severe and can allow the model to give more human-like predictions for better decision making.^4^ A common set of approaches used in machine-learning UQ are Bayesian methods,^5^ for example, applying Monte Carlo inference^6^ to a model for stroke diagnosis^7^ or model ensemble methods^8^ applied to brain tissue microstructure measurement.^9^ Common uses of UQ are out-of-distribution detection, quality control, and calibration.^10^

Beyond using UQ for model understanding and decision making, UQ can be integrated directly into training to produce a measure of aleatoric uncertainty as an additional model output^11^ or by replacing standard loss functions with uncertainty-aware alternatives, such as substituting cross-entropy loss with focal loss*.*^12^ In active learning, Bayesian UQ methods can be used to select and train on highly uncertain images for classification,^13^ in semi-supervised learning^14^ for pseudo-label selection,^15^ and to create uncertainty-based pseudo-labels for semi-supervised segmentation.^16^

UQ can also be integrated directly into model architectures, with U-QNet incorporating a feature pyramid attention module into the U-Net structure to quantify uncertainty in prostate segmentation.^17^ By combining UQ and state-of-the-art segmentation models, MedSAM-U^18^ achieves better uncertainty-led prompt selection and U-MedSAM creates a sub-task specific (pixel, region, and distribution), uncertainty-weighted loss function.^19^

Conformal prediction has gained popularity as a model-agnostic, post hoc, and distribution-free method of UQ that can be used with any model.^2^ For example, conformal prediction can be applied to grading the severity of spinal stenosis in lumbar spine magnetic resonance imaging (MRI) scans to increase trust in model predictions.^20^ By exploring the relationship between uncertainty, as measured by conformal prediction set size and model performance, more nuanced clinical decisions can be made, for instance, in a cervical-screening pipeline.^21^ Conformal prediction has also shown superior results for identifying out-of-distribution examples.^22^

Our contribution to this line of research is to present a comparison of several lightweight UAT pipelines using output-level uncertainty, Bayesian methods in the form of the variance of model outputs, and conformal prediction to improve segmentation outcomes in three single-class (background and structure) segmentation datasets. We note here that we are not attempting to outperform state-of-the-art models, but to demonstrate how simple uncertainty map additions to our chosen loss function improves model performance without changing model convergence or complexity appreciably.

## Methods

### Model

For the foundational model to fine-tune, we used the Medical Segment Anything Model^18^ (MedSAM), a segmentation model based on Segment Anything Model^23^ but fine-tuned on medicine-specific tasks. As the original MedSAM model requires prompts, we left the prompt as the entire image to force the model to find the segmentations without guidance.

### Uncertainty Quantification

We apply three different UQ methods to generate uncertainty maps, entropy, conformal prediction,^2^ and what we are calling variance, which functions similarly to Monte Carlo methods.^24^ Each of these gives us an uncertainty map *U* ∈ R^*B*×*H*×*W*^.

#### Entropy

We calculate the entropy per pixel:

$$\begin{array}{c} U=-\hat{Y}\log\left(\hat{Y}\right)-\left(1-\hat{Y}\right)\log\left(1-\hat{Y}\right) \end{array}$$

where $\hat{Y}$ is the predicted probability map of shape *C* × *H* × *W*, that is, per-pixel probability distribution across *C* classes.

#### Conformal Prediction Overview and Least Ambiguous Set-Valued Classifier

For classification, the goal is to develop a model, ${\hat{f}}_{y}\left(x\right)$, which estimates P(Y=y|X=x), where *y* is the label and *x* is the datum, with outputs in Δ^*K*^, the *K*-simplex, a valid probability distribution.

We will construct a conformal prediction set for a test point *x_test_*, $\hat{C}\left(x_{test}\right)\subseteq Y$, where Y is all our possible classes (i.e. |Y|=K), such that $P[y_{test}\in \hat{C}\left(X_{test}\right)]$. We call 1 − α the (empirical) coverage and α is the error rate.^2^

In this work, we used the specific conformal prediction algorithm Least Ambiguous Set-Valued Classifier (LAC).^2^

We will divide our data X into three sets, $X_{train}$, $X_{calibration}$, and $X_{test}$, where $X_{train}$ is our standard training set used to train the model $\hat{f}$, $X_{calibration}$ is a calibration set to prepare for our conformal predictions, and $X_{test}$ is the set of data we wish to construct conformal predictions for. Let *n_cal_* be the number of calibration points.

Now, we introduce a score function, *s*(*x*, *y*) which tells us how well the model is performing. The LAC algorithm uses the probability of that specific class. As in, if $\hat{f}{\left(x\right)}_{y}=\left[p_{0},...,p_{K-1}\right]$, we can take:

$$\begin{array}{c} s\left(x,y\right)=\;1-\hat{f}{\left(x\right)}_{y_{i}},\;y_{i}=\;\mathrm{Index}\;\mathrm{of}\;\mathrm{correct}\;\mathrm{class}. \end{array}$$

For each element of our calibration set $X_{cal}$, we repeat the above process, giving us $\left\{s_{1},...,s_{n_{cal}}\right\}$, from which we calculate the quantile:

$$\begin{array}{c} \hat{q}=quantile\left(\left\{s_{1},...,s_{n_{cal}}\right\};\frac{\left\lceil \left(1-\alpha \right)\left(n_{cal}+1\right)\right\rceil }{n_{cal}}\right) \end{array}$$

From this, we construct our $\hat{C}\left(x_{test}\right)$ as:

$$\begin{array}{ccc} & & \;\hat{C}\left(x_{\mathrm{test}}\right)\\ & & \;=\left\{\;y:s\left(x_{\mathrm{test}},y_{\mathrm{test}}\right)\leq \hat{q}\;\right\}=\left\{\;y:{\hat{f}}_{y}\left(x_{\mathrm{test}}\right)\geq 1-\hat{q}\;\right\}. \end{array}$$

Since we don't have *y_true_* for our test point, we are choosing all the indices of $\hat{f}\left(x_{test}\right)$ with a value greater than $1-\hat{q}$.

As this is a post hoc method of uncertainty quantification, we cannot render an uncertainty map immediately or, if we did, the calibrations would be meaningless. Instead, we allow the models to learn normally for *n_warmup_* runs before beginning to apply conformal prediction. After *n_warmup_* runs, the $\hat{q}$ is calculated and conformal uncertainty maps are generated. We note here that we *also* apply this warmup period to the other UAT pipelines for the same reason, to allow the models to learn a bit before forcing them to consider their uncertainty and we apply this at the next epoch. For the conformal-based UAT, this same value serves as the recalculate interval, updating the $\hat{q}$ every *n_warmup_* epochs.

#### Variance

Natively, MedSAM models provide three outputs per datum unless a keyword argument is set. With this off, we retain three outputs. We calculate the per-pixel variance^25^ of these three outputs as the uncertainty map for that datum.

$$\begin{array}{c} U=\frac{1}{M}\sum_{m=1}^{M}{\left({\hat{Y}}_{m}-\bar{Y}\right)}^{2},\;\bar{Y}=\frac{1}{M}\sum_{m=1}^{M}{\hat{Y}}_{m}. \end{array}$$

### Loss

To incorporate uncertainty information into training, we use a weighted Binary Cross-Entropy (BCE) loss formulation, where pixel-wise uncertainty maps modulate the contribution of each pixel to the total loss. This allows the network to focus more heavily on uncertain or ambiguous regions. Let *U* be the uncertainty map. We define a non-decreasing weight *W_i_*  =  1  +  γ *U_i_*,γ  ≥  0. Thus, no pixel is down-weighted (*W_i_* ≥ 1), while more uncertain pixels receive proportionally larger loss contributions. The resulting uncertainty-weighted BCE is

$$\begin{array}{c} L_{\mathrm{wBCE}}\;=\;-\;\frac{1}{N}\sum_{i=1}^{N}W_{i}\left[\;Y_{i}\log{\hat{Y}}_{i}\;+\;\left(1-Y_{i}\right)\log\left(1-{\hat{Y}}_{i}\right)\;\right] \end{array}$$

with *N* the number of pixels. When γ = 0, *W_i_* = 1 and the above reduces to the standard BCE. As γ increases, pixels with higher *U_i_* are emphasized, encouraging the model to concentrate on ambiguous regions (e.g., lesion borders).

To summarize the various uncertainty types, entropy measures how uncertain the model is about its own predictions, variance measures how much the model's predictions change when it is run multiple times, and conformal prediction measures how unusual a new prediction is compared to what the model has learned to expect.

### Data Description

We apply the same training pipelines to three single-class ophthalmic segmentation datasets. These are GA, OC, and FAZ segmentation tasks.

The GA dataset is an in-house dataset of 110 fundus autofluorescence (FAF) images from 73 patients with hand-annotated GA maps from a single ophthalmologist in a 50/40/10/10 train/test/calibration/validation split.

The OC dataset is the PAPILA dataset^26^ consisting of 438 full-color fundus images in a 238/146/51/51 train/test/calibration/validation split with annotations for optic disc and OC provided by two specialists. The STAPLE algorithm^27^ was used to combine the two annotations into a single one for segmentation purposes. The optic cup was chosen from these annotations as the more difficult case to apply UAT to.

The FAZ data are from the OCTA500 dataset,^28^ a publicly available collection of paired optical coherence tomography (OCT) and optical coherence tomography angiography (OCTA) volumes acquired using AngioVue scanners. The dataset contains 478 eyes (normal, diabetic, and high-myopia) across 3 × 3 mm and 6 × 6 mm fields of view.^29^ For this work, we used the provided 2D FAZ projections in a 150/90/30/30 train/test/calibration/validation split.

## Experiment

We evaluate three UAT strategies, LAC conformal prediction with α∈{0.05,0.10,0.15,0.20,0.25, 0.30}, entropy, and model variance, as well as a standard finetuning without UAT, the baseline. Each configuration was run 10 times with the same dataset splits to account for training stochasticity. Training is guided by early stopping using unweighted loss with a patience of 5 epochs, using a maximum of 40 epochs to ensure convergence, and is kept consistent with Adam^30^ weight optimizer with a learning rate and weight decay hyperparameters of 1  × 10^−5^ and the rest default. Due to memory constraints, we used a batch size of 1. Preprocessing consists of applying the accompanying MedSAM preprocessing function. As MedSAM requires prompts, we set the prompt to the entire image to simulate not having any human-level guidance. During training, we record the model outputs and uncertainty maps for the first batch of the training and validation sets of each epoch to evaluate how they evolved over time.

Finally, we run a hyperparameter search over the warmup period $n_{warmup}\in \left\{1,\;2,\;3,\;4,\;5\right\}$ epochs and the γ∈{1,2,3,4,5}. The rationale for ranging over different warmup periods is that adding uncertainty too early might result in the models focusing on unimportant, but highly uncertain, regions, hurting performance. Similarly, fine-tuning the strength of the γ hyperparameter aims to find the balance point between the models not considering uncertain regions enough, reducing the power of UAT, and being led into focusing too much on potentially unimportant areas. The nonparametric Mann-Whitney *U* test^31^ was used to determine statistically significant differences in UAT models’ performances versus the non-UAT baseline. Further, Levene's test for equality^32^ was used to determine whether UAT helped make the trainings more stable, as we see large training instability in the non-UAT baseline.

Our hypotheses for this experiment are that at least one, but likely more, UAT pipelines improve average performance in each dataset in comparison to the regular, non-UAT, training pipeline and improve training stability. In doing so, we expect to achieve faster average training as measured by the models converging earlier.

## Results

### Hypothesis: UAT Improves Performance and Training Stability

**Figure 1.**
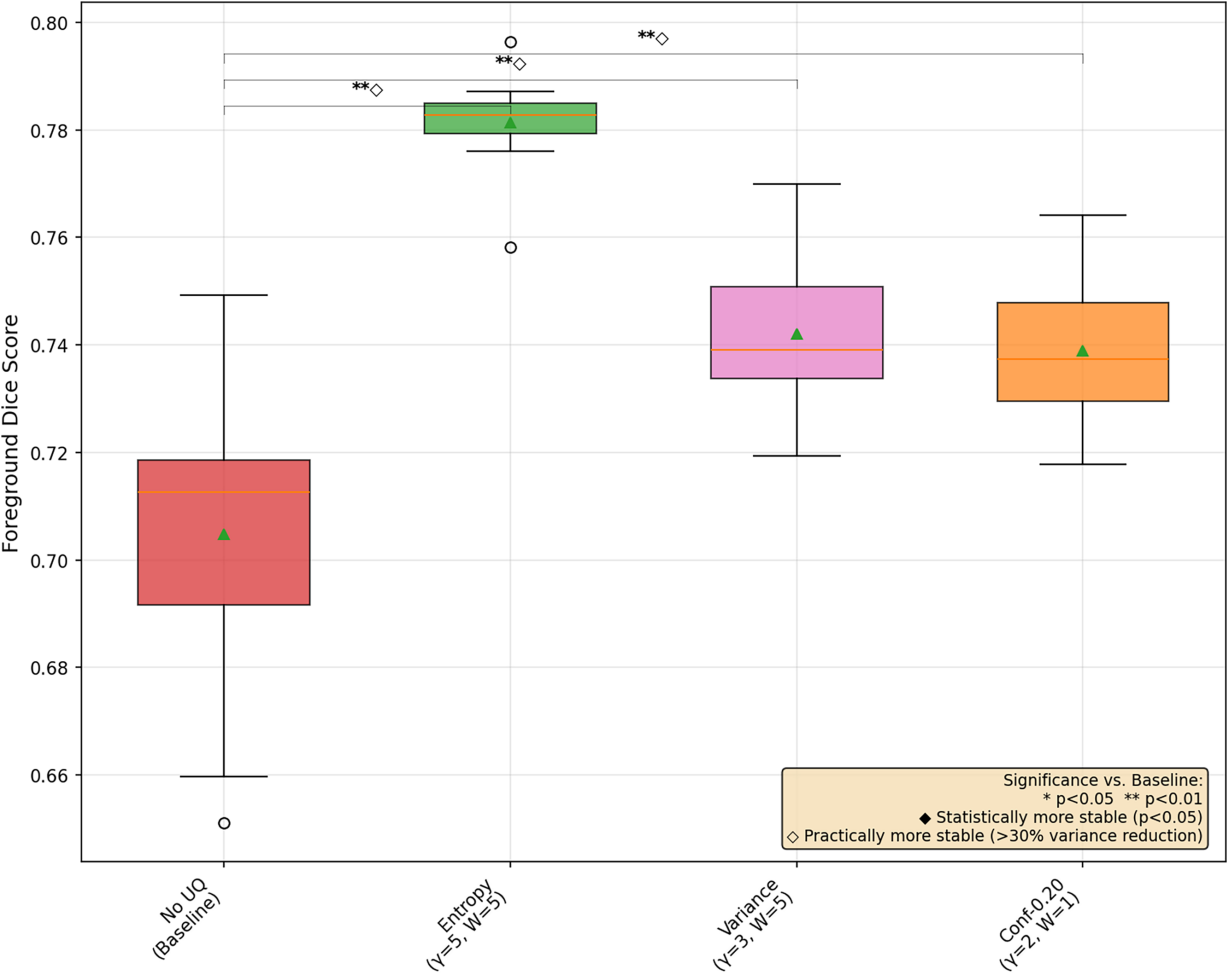
Box-and-whisker plot of three UAT pipelines and a base training for OC segmentation with MedSAM.

**Figure 2.**
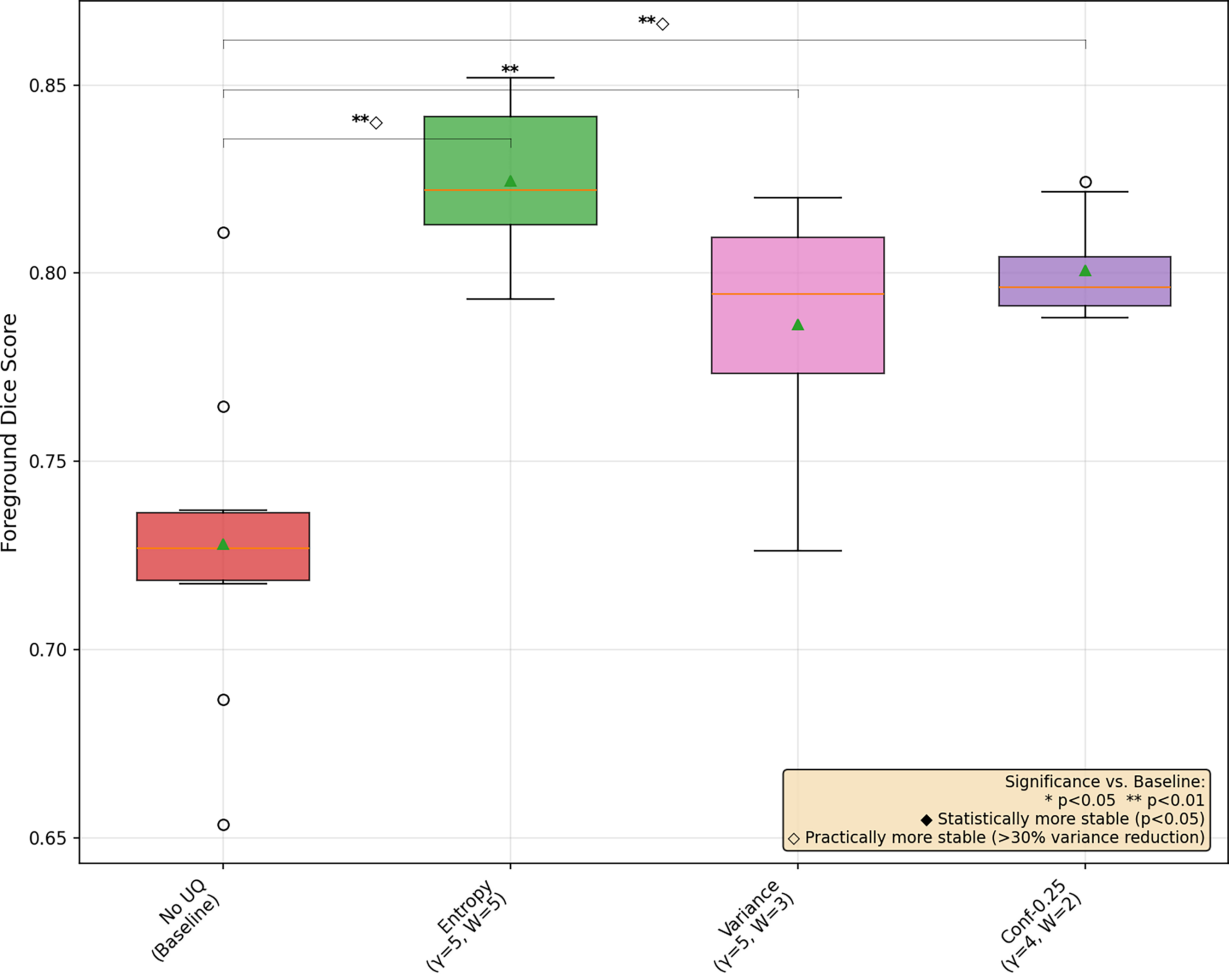
Box-and-whisker plot of three UAT pipelines and a base training for GA segmentation with MedSAM.

**Figure 3.**
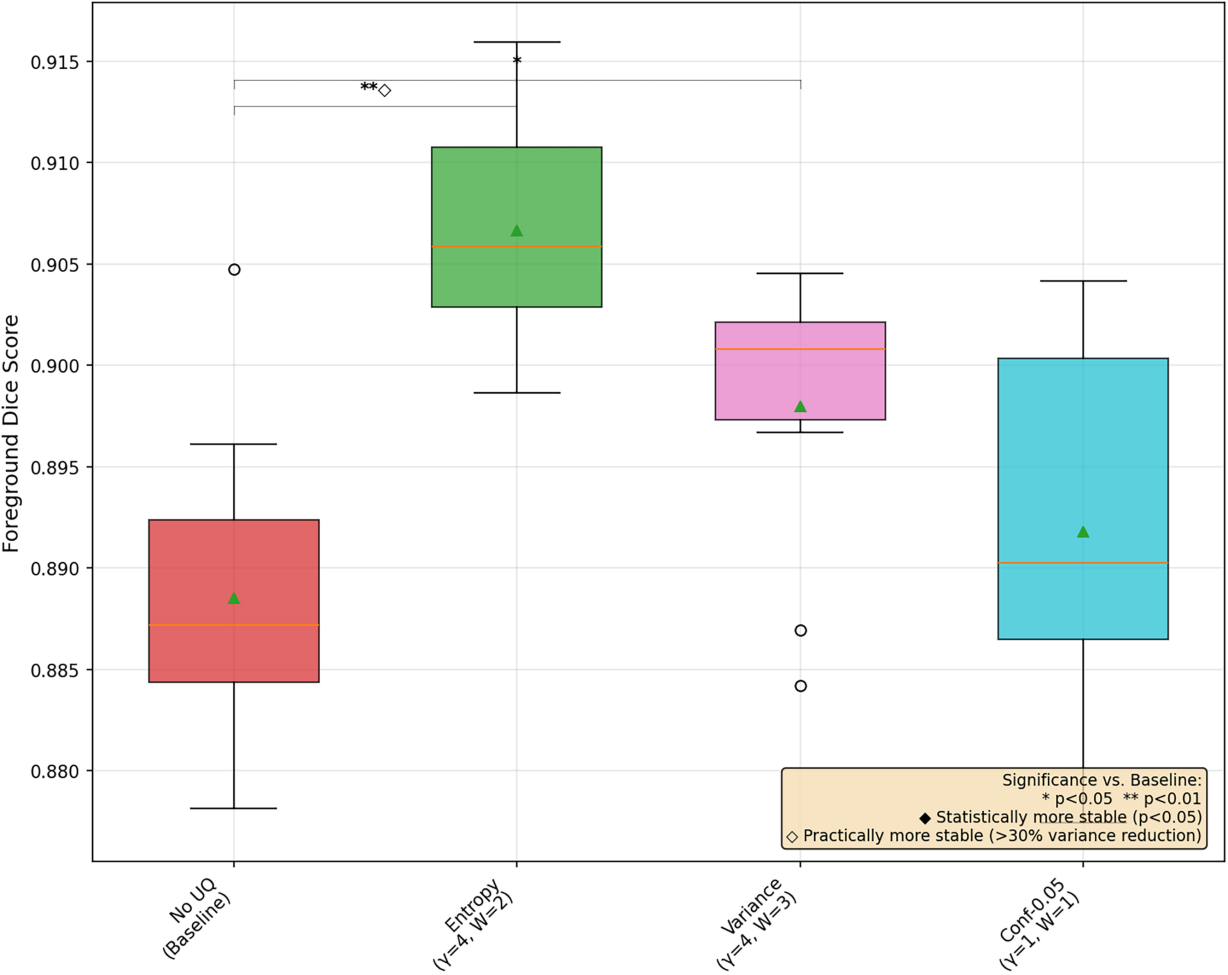
Box-and-whisker plot of three UAT pipelines and a base training for FAZ segmentation with MedSAM.

In Figures 1, 2, and 3, we provide a box-and-whisker plot of the Dice scores for each dataset over the 10 runs of best UAT and non-UAT pipelines, choosing only the best α values among the conformal prediction-based UAT pipelines. In these graphics, we see similar patterns of performance; entropy provides statistically significant improvements in model performance, and although it does not reach the level of statistical significance at the *P* < 0.05 level, there is a clear, large decrease in the variance of the model performances in each dataset. We also note surprisingly that the best conformal prediction-based UAT are similar with α = 0.20 and 0.25 for OC and GA respectively, and α = 0.05 for the FAZ. However, for FAZ, this potential improvement in performance by the conformal prediction-based UAT is not significant.

**Table.** Convergence Speed Comparison: Average Training Epochs vs. Baseline

Dataset	Method	Avg. Epochs	Difference (%)	*P* Value
Cup	Baseline	8.5	—	—
Cup	Conf-0.05	7.1	−16.5%	0.021*
Cup	Conf-0.10	8.1	−4.7%	0.608
Cup	Conf-0.15	8.2	−3.5%	0.607
Cup	Conf-0.20	9.0	+5.9%	0.331
Cup	Conf-0.25	8.8	+3.5%	0.815
Cup	Conf-0.30	8.2	−3.5%	0.559
Cup	Variance	9.0	+5.9%	0.744
Cup	Entropy	9.8	+15.3%	0.084
GA	Baseline	12.0	—	—
GA	Conf-0.05	5.0	−58.3%	0.150
GA	Conf-0.10	12.8	+6.7%	0.909
GA	Conf-0.15	10.8	−10.0%	0.158
GA	Conf-0.20	14.1	+17.5%	0.108
GA	Conf-0.25	21.4	+78.3%	0.004*
GA	Conf-0.30	16.0	+33.3%	0.254
GA	Variance	11.0	−8.3%	1.000
GA	Entropy	12.4	+3.3%	0.909
FAZ	Baseline	17.9	—	—
FAZ	Conf-0.05	9.4	−47.5%	<0.001***
FAZ	Conf-0.10	15.2	−15.1%	0.129
FAZ	Conf-0.15	15.2	−15.1%	0.093
FAZ	Conf-0.20	13.9	−22.3%	0.005**
FAZ	Conf-0.25	15.9	−11.2%	0.321
FAZ	Conf-0.30	13.5	−24.6%	0.020*
FAZ	Variance	13.0	−27.4%	0.150
FAZ	Entropy	19.9	+11.2%	0.595

* *P* < 0.05, ** *P* < 0.01, ***P < 0.001.

Bold values indicate statistically significant differences. Negative percentages indicate faster convergence.

### Hypothesis 2: UAT Results in Faster Training

To determine if the addition of uncertainty maps in the loss calculation results in quicker training times, the same Mann-Whitney *U* test was used on the number of epochs required for convergence, and the results are more mixed, as demonstrated in Table. The FAZ has several UAT pipelines converging faster in a statistically significant way, but not the best UAT, entropy. For GA and OC, few UAT pipelines make an appreciable difference in convergence epochs at a statistically significant level.

### Conformal Prediction Ablations

The choice of α value to determine the coverage in conformal prediction is not an obvious one. Small α values result in higher coverage, but larger average prediction set sizes. Thus, a balance needs to be struck between coverage and prediction set size, as an α value too small leads to too many unimportant pixels being highlighted and having a value too large leads to very few uncertain pixels. In the following ablation studies, we compare LAC conformal prediction-based UAT with the following α values: 0.05,  0.1,  0.15,  0.2,  0.25, and 0.3. We show in each case the best γ and *n_warmup_* version.

**Figure 4.**
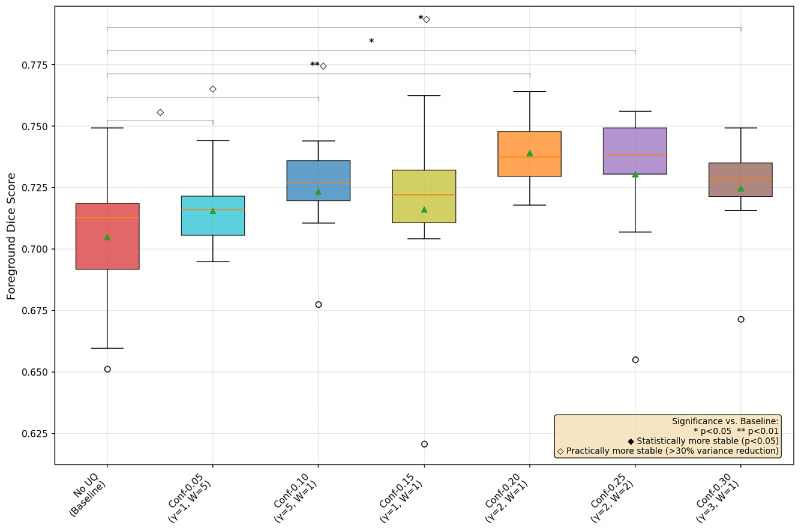
Box-and-whisker plot of conformal prediction-based UAT OC segmentation with all ***α*** values.

**Figure 5.**
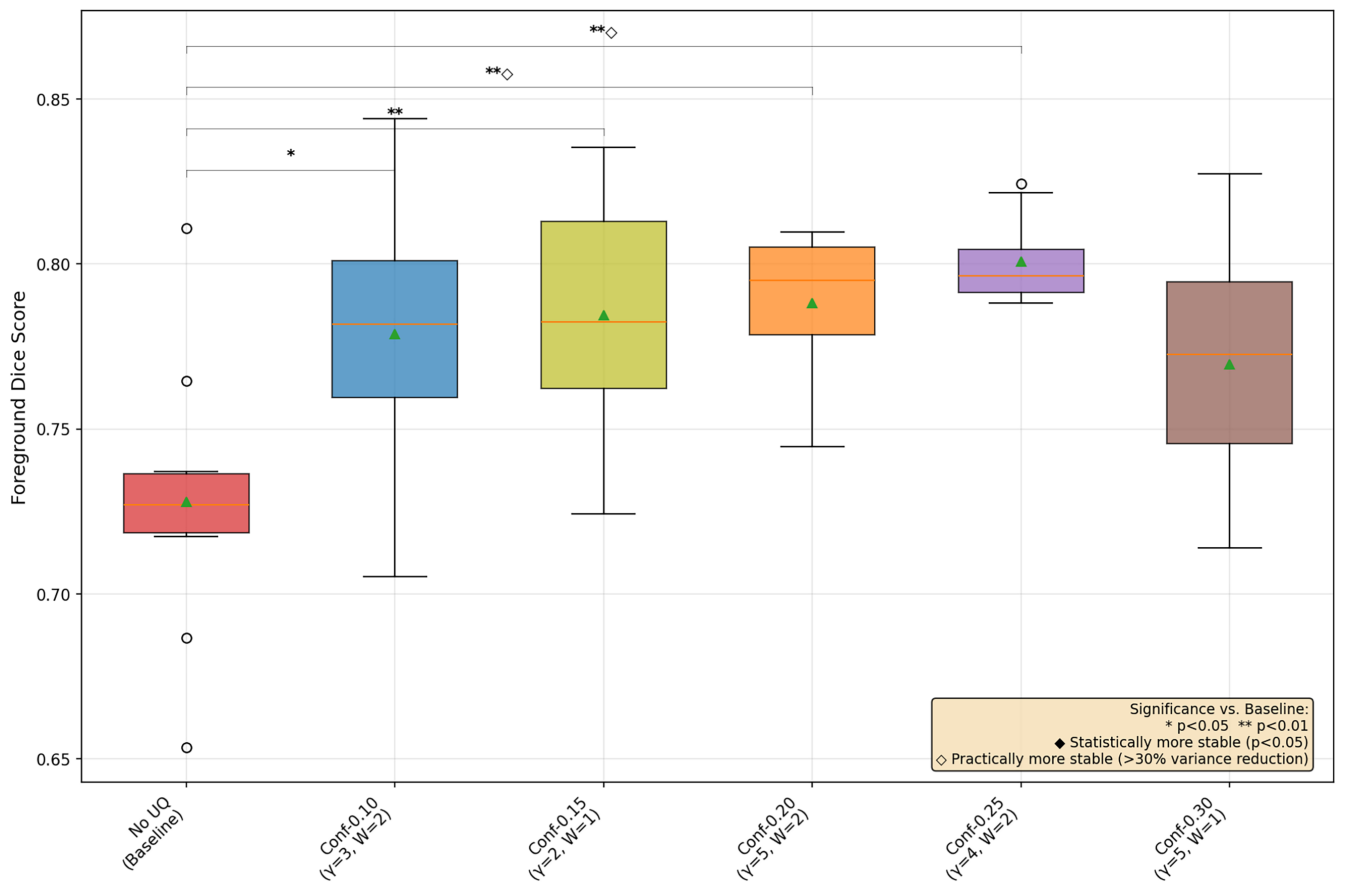
Box-and-whisker plot of conformal prediction-based UAT GA segmentation with all ***α*** values.

**Figure 6.**
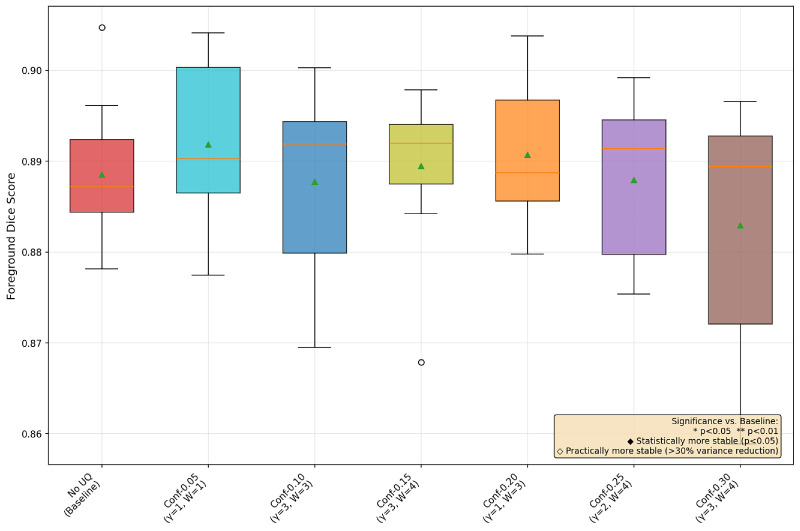
Box-and-whisker plot of conformal prediction-based UAT FAZ segmentation with all ***α*** values.

From Figures 4, 5, and 6, we see that the OC and GA datasets behave similarly, with the conformal-based UAT generally helping with both performance and training stability most around the α = 0.20/0.25 range. However, for the FAZ segmentation task, none of the conformal-based UAT pipelines make much difference to performance nor training stability. Speculating as to why these α values do better, we again reiterate the trade-off between having larger areas of uncertainty versus confusing the model by forcing it to focus on unimportant areas, with α smaller than 0.15 highlighting too many unimportant regions and greater than 0.25 creating almost no uncertain regions, allowing the model to essentially default back to the non-UAT, baseline training.

### Uncertainty Throughout Training

**Figure 7.**
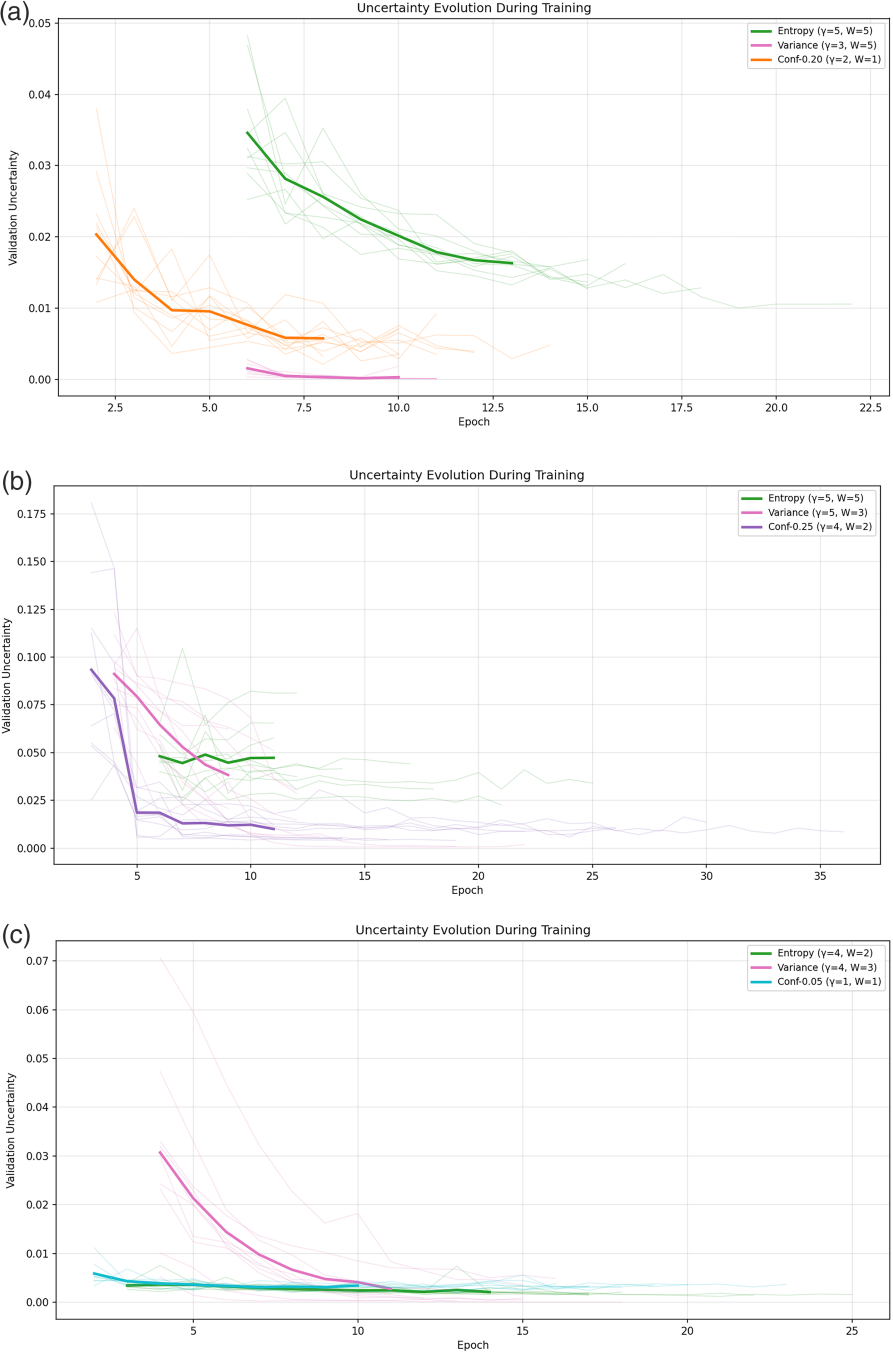
(**a**) Uncertainty magnitude across training epochs for the best-performing UQ configurations for the OC segmentation task. (**b**) Uncertainty magnitude across training epochs for the best-performing UQ configurations for the GA segmentation task. (**c**) Uncertainty magnitude across training epochs for the best-performing UQ configurations for the FAZ segmentation task. Uncertainty changes across tasks. Average is in bold and individual runs are in the same, lighter color for the that task's best UQ method. (**a**) OC, (**b**) GA, and (**c**) FAZ.

In Figure 7, we plot the uncertainty across epochs for the best-performing models, with the bold lines being an average over the shortest-converging model run and the lighter colors being individual runs.

From Figure 7, we see that the uncertainty decreases over training epochs, with seemingly three exceptions, the entropy for GA in Figure 7b, which remains more stable, although it does appear to decrease in some runs (in light green), and entropy and conformal with α = 0.05 in the FAZ segmentation in Figure 7c which both hover around 0. Although the results on the FAZ are surprising, as the entropy-based UAT does lead to statistically significant performance improvements, it is non-zero, albeit small, throughout training, indicating that there is still some uncertainty to be captured by this method that the models can exploit.

### Examples of Uncertainty Across Training Epochs

**Figure 8.**
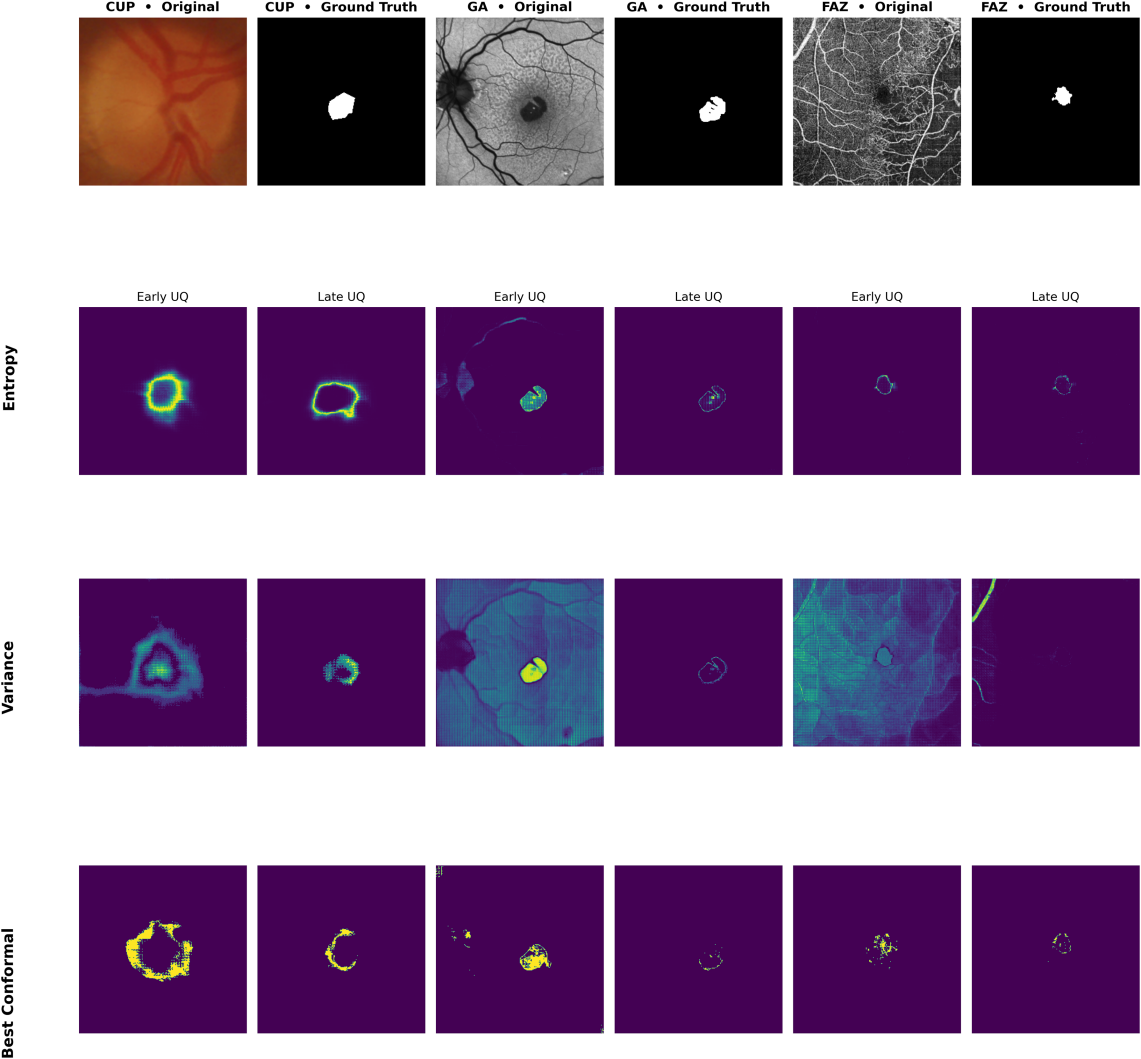
Examples of each segmentation task's uncertainty for the best models for each dataset.

To observe the change in uncertainty and segmentation outcomes during the training process, Figure 8 shows example uncertainty maps during UAT and the associated segmentations at various epochs of the best-performing models of each UAT type. “Early” will refer to the first epoch in which the uncertainty map is calculated, depending on the warmup period. For space considerations, we do not include this value (nor the γ parameter) as these are the same best runs described earlier. “Late” refers to the last epoch of training, which will vary for each pipeline. Each image is taken from the first run in the first batch for consistency.

In each of these, we can see that while the uncertainty decreases, as expected from Figure 7, the uncertainty from the entropy UAT pipeline more closely aligns with where it is expected, at the edges of the regions in question, and crucially remains present even by the end of training, highlighting how this uncertainty is capturing areas where the models struggle. Further, although the conformal seems to follow this pattern early, by later epochs, this method is not capturing much uncertainty at all, meaning that the model benefits less as training progresses using conformal-based UAT. Finally, the variance, although it seems to follow the pattern established by entropy in the case of OC segmentation, for the GA and FAZ segmentation tasks, it is essentially 0 by the end, meaning loss has reverted back to being unweighted (or weighted by 1).

## Discussion

From the previous results, we can state clearly that for all three datasets, entropy-based UAT improved model performance at a statistically significant level. In the GA and OC segmentation tasks, variance in MedSAM model outputs and conformal prediction with the LAC algorithm with α values approximately 0.20 also improved model performance. Further, in the GA and OC segmentation tasks, UAT generally improved training stability, not at a statistically significant level, but greater than 30% compared to the non-UAT training. Entropy-based UAT also provided this advantage for the FAZ segmentation task. However, in general, although the performance and training stability increased, the convergence times remained relatively unchanged.

However, Figures 7 and 8 highlight a key trade-off in applying UAT. Larger uncertainty regions that fail to align with model errors provide little benefit, whereas overly small regions, such as those observed with conformal-based UAT and variance weighting, lead the loss function to behave almost identically to its unweighted form once uncertainty diminishes. Task difficulty also matters; easier and more deterministic problems, such as FAZ segmentation, gain little from UAT, whereas more complex tasks like OC and GA segmentation show clear improvements.

As a practical advantage, entropy-based UAT is simple to implement, architecture-agnostic, and adds no model complexity, making it an attractive enhancement for many pipelines. However, potential drawbacks remain. Poorly tuned uncertainty weighting may emphasize irrelevant regions and degrade learning, and hyperparameters such as γ and warmup intervals require empirical tuning to balance stability and sensitivity. Whereas this adds computational overhead, the consistent performance gains suggest that, when resources allow, incorporating UAT offers a low-cost path toward more robust segmentation. In addition to improving training stability, the UAT-produced uncertainty maps found in Figure 8 serve as intuitive visualizations of model confidence. These maps can help identify ambiguous cases or inconsistent labels and provide clinicians with a measure of trust in the model's outputs. Thus, even when UAT does not significantly improve quantitative metrics, it adds interpretability that can benefit downstream use.

### Next Steps

Despite these successes, several questions remain. All three tasks presented here are single-class segmentations, and the performance on multi-class segmentation is a natural next step. Further, different conformal prediction algorithms can be easily added to this analysis in the future and other output-level UQ methods similar to entropy, such as the variance of the probabilities or margin-based uncertainty, can be calculated directly from the output probability maps and do not need a calibration step. These can help with the limitations of this work, which include small datasets (e.g., GA) that do not allow for all uncertainty methods to be used, the need to fine-tune the γ and warmup epochs parameters, the minimal gain in datasets where the baseline already performs well, and that the uncertainty pixels do not always align with error. Increases in the data and number of datasets, kinds of uncertainty quantification, and incorporating multi-class segmentation will help outline the situations in which different UAT types can be expected to outperform the baseline and provide more robust statistical evidence. Finally, extending UAT to more loss functions will determine the cost landscapes in which UAT is most effective.

## Conclusions

This study demonstrates that incorporating model uncertainty directly into the loss function improves segmentation performance and training stability without increasing model complexity. Our goal was not to achieve state-of-the-art results, but to test the feasibility of integrating uncertainty weighting into standard loss functions such as binary cross-entropy. Whereas Dice loss remains a strong baseline, it is less naturally compatible with this form of weighting, motivating future work on hybrid or uncertainty-aware variants of Dice-based losses.

Translationally, these findings provide early evidence that uncertainty-aware training can make segmentation models more reliable by aligning model learning with regions of inherent uncertainty. When uncertainty and error coincide, this approach improves performance without slowing convergence. Future work will extend these analyses to additional loss functions and datasets, advancing the integration of uncertainty into deep learning pipelines for ophthalmic image analysis.
